# Eugenyl-2-Hydroxypropyl Methacrylate-Incorporated Experimental Dental Composite: Degree of Polymerization and In Vitro Cytotoxicity Evaluation

**DOI:** 10.3390/polym14020277

**Published:** 2022-01-11

**Authors:** Abdel-Basit Al-Odayni, Dalal H. Alotaibi, Waseem Sharaf Saeed, Abdullah Al-Kahtani, Ali Assiri, Fahad M. Alkhtani, Ali Alrahlah

**Affiliations:** 1 Engineer Abdullah Bugshan Research Chair for Dental and Oral Rehabilitation, College of Dentistry, King Saud University, Riyadh 11545, Saudi Arabia; wsaeed@ksu.edu.sa (W.S.S.); aalrahlah@ksu.edu.sa (A.A.); 2 Department of Periodontics and Community Dentistry, College of Dentistry, King Saud University, Riyadh 11545, Saudi Arabia; dalalotaibi@ksu.edu.sa; 3 Chemistry Department, College of Science, King Saud University, Riyadh 11451, Saudi Arabia; akahtani@ksu.edu.sa; 4 College of Dentistry Research Center (CDRC), College of Dentistry, King Saud University, Riyadh 11545, Saudi Arabia; benmerzin@gmail.com; 5 Department of Prosthodontics, College of Dentistry, Prince Sattam Bin Abdulaziz University, Alkharj 11924, Saudi Arabia; f.alkhtani@psau.edu.sa; 6 Restorative Dental Sciences Department, College of Dentistry, King Saud University, Riyadh 11545, Saudi Arabia

**Keywords:** eugenol derivative, polymerizable eugenol, dental composite, cytotoxicity

## Abstract

The aim of this study was to evaluate the properties of new dental formulations containing eugenyl-2-hydroxypropyl methacrylate (EgGMA) monomer, as restorative dental material, in terms of their degree of photopolymerization and cytotoxicity. The target model composites (TBEg0, TBEg2.5, and TBEg5) were prepared by mixing 35% organic matrix (TEGDMA/BisGMA (50/50 wt%) of which 0, 2.5, and 5 wt%, respectively, were replaced with EgGMA monomer) with 65% filler (silanized hydroxyapatite (HA)/zinc oxide (ZnO_2_), 4:3 by weight). The vinylic double-bond conversion (DC) after light-curing was studied using Fourier transform infrared technique whereas cell viability was in vitro tested using primary human gingival fibroblasts cells over 7 days by means of AlamarBlue colorimetric assay. The obtained data were statistically analyzed using ANOVA and Tukey post-hoc tests. The results revealed no significant difference in DC between TBEg2.5 (66.49%) and control (TBEg0; 68.74%), whereas both differ significantly with TBEg5, likely due to the inhibitory effect of eugenol moiety at high concentration. The cell viability test indicated that all the composites are biocompatible. No significant difference was counted between TBEg2.5 and TBEg5, however, both differed significantly from the control (TBEg0). Thus, even though its apparent negative effect on polymerization, EgGMA is potentially safer than bisphenol-derived monomers. Such potential properties may encourage further investigations on term of EgGMA amount optimization, compatibility with other dental resins, and antimicrobial activity.

## 1. Introduction

Eugenol (Eg) is a natural essential oil, the major component of the dried flower buds of the clove tree, used beneficially in various applications and for several purposes. Since the ancient times, it has been used as an antimicrobial and antiseptic agent. Nowadays, it is avowed to possess certain properties as a nutraceuticals and pharmaceutical ingredient, including anesthetic activity, antioxidant potential, antimicrobial role, anti-inflammatory action, anti-carcinogenic effects, neuroprotective ability, hypolipidemic efficiency and anti-diabetic effectiveness [1]. The functionality of Eg is proposed to be due to the presence of phenol, and allylic groups [2]. As a result of its bioactivity, it has been involved in a wide range of biomedical applications, including dentistry.

However, despite its desirable properties as an analgesic, the incorporation of Eg (free molecule) in dental resin composites is not recommended, because of its negative effect on the overall properties of the composites, including degree of double bond-conversion (DC), water solubility, and mechanical properties [3,4]. Additionally, eugenol is a strong-smelling and volatile compound, which is another reason for the undesirability of its usage in dental composites. However, Eg in combination with zinc oxide (ZnO_2_) constitutes a dental formulation commonly used both as a luting and temporary restorative material. Due to its low strength and high oral solubility, ZnO_2_–eugenol (ZOE) is not recommended as a permanent luting cement [5]. On the other hand, eugenol (the free molecule) is not compatible with other methacrylate-based restorative materials because of its inhibitory effect on the polymerization process of dental composite resins [6].

Various derivatives of eugenol, targeting its hydroxyl functional group as a reacting site for production of, for example, esters or ethers with different substituents, have been prepared, and their properties, including antimicrobial and cytotoxicity, have been documented [7,8,9]. For instance, Rahim et al. [9] prepared a series of eugenol derivatives, evaluated their antibacterial activity against a range of bacteria strains, and reported a derivative-dependent activity with noticeably high, broad-spectrum antibacterial activity of eugenyl *p*-bromobenzoate. However, many of these derivatives, ethers in particular, showed no or comparable activity compared to that of eugenol. Martins et al. [7], on the other hand, evaluated the antimicrobial and cytotoxicity characteristics of eugenol analogs prepared via acylation or alkylation methods (ester and ether products). The antibacterial activity was again observed to be bacterial and substituent selective. The overall cytotoxicity test against NIH/3T3 fibroblast cell line (NIH Swiss 3-day transfer, inoculum 3 × 10^5^ cells) performed using 3-(4,5-dimethylthiazol-2-yl)-2,5-diphenyltetrazolium bromide (MTT) assay revealed no cytotoxic effect on the tested normal cell line, demonstrating the ability to selectively kill microorganism cells with no or little damage to normal cells.

Conversion of Eg into polymerizable derivatives may be one solution to its unfavored properties, such as its strong odor and high volatility, for dental use. In this context, a number of polymerizable Eg-derivates have been synthesized [10,11,12], and their physical, chemical, mechanical and biological properties have been reported. Almaroof et al. [12] investigated various properties of dual-cured dental composites incorporating eugenyl methacrylate (EgMA) as in post and core build-up restoration. However, the polymerization degree of conversion, curing depths, and exotherm were decreased with respect to the increase in EgMA. Moreover, an experiment concerning the formulation of various polymerizable Eg-derivatives for dental and orthopedic applications were conducted by Rojo et al. [6], who reported enhanced cement properties, including mechanical and bactericidal effect against certain bacteria strains. Such behaviors may be a result of the crosslinking reaction due to the participation of the allylic group present in the eugenol derivatives [13]. Generally, the favored properties of any dental composite additive are its ability to demonstrate an effective antimicrobial activity without compromising cytotoxicity on human cells, and other desirable properties [14].

The cytotoxicity of a dental composite depends on the chemical composition, the type and amount of leached component, and the leached medium [15]. When new materials are developed, the toxicity of the final polymerized materials, and their physical and chemical properties are scrutinized. For monomers, in particular, structure–toxicity relationship is one interesting matter to be evaluated, after which a particular application could be affirmed [16]. Studies concerning structure–toxicity relationship, including hydrophilicity–lipophilicity, have reported a reasonable correlation between the hemolytic activity of monomers and their chemical structure; therefore, the toxicity of BisGMA is due to its hydrophobicity, which promotes its affinity for erythrocytes. However, the cytotoxicity of dental monomers has been reported to decrease in the following order: BisGMA > UDMA > UDMA > TEGDMA > HEMA > MMA [17,18].

In this work, a new immobilizable methacrylate-based derivative of eugenol (EgGMA) was synthesized, and its benefits for dental application were evaluated. Therefore, resin-based restorative composites were prepared, incorporating 2.5 and 5 wt% EgGMA monomers. The main objectives were to report on the cytotoxicity of the composites against human fibroblast cells, and on the monomer effects on the final properties, including DC of the composite, employing the relevant advanced techniques in the investigation.

## 2. Materials and Methods

### 2.1. Materials

The base resins BisGMA (>98%) and TEGDMA (>95%), photo-initiator camphorquinone (CQ, 97%), curing accelerator 2-(dimethylamino)ethyl methacrylate (DMAEMA; 98%)), coupling agent (3-(trimethoxysilyl)propyl methacrylate (γ-MPS, 98%), filling materials (hydroxyapatite (HA, ≥97%) and zirconium dioxide (ZrO_2_, 99%)), reaction reagents eugenol (Eg, 98.5%) and glycidyl methacrylate (GMA, 98%), and the radical polymerization inhibitor hydroquinone (HQ, >99%) were purchased from Sigma–Aldrich (Taufkirchen, Germany). The reaction catalyst triphenylphosphine (Ph_3_P, 99%) was procured from Cica-reagent (Kanto Chemical, Tokyo, Japan). The solvents used ethyl acetate (EA, 99.5%) and *n*-hexane (*n*-Hx, 95%) were obtained from Fisher Scientific (Loughborough, UK).

### 2.2. EgGMA Synthesis

Eugenyl-2-hydroxypropyl methacrylate (EgGMA) was synthesized using a previously reported method [19]. Briefly, equimolar amounts of the reactants, Eg and GMA, were homogenized, with stirring, in a three-necked round-bottom flask. To this solution, 0.5 wt% and 0.1 wt%, respectively, of HQ (an inhibitor used to prevent vinyl and allyl polymerization possibility under the applied reaction condition) and Ph_3_P (a catalyst applied to facilitate ring opening-based etherification reaction) with respect to the total weight of monomers were added. The reaction was performed under nitrogen atmosphere with reflux at 120 °C for 2 h. Thin-layer chromatography (TLC) was used for monitoring the reaction completion and further purity assessment (aluminum plate, silica as stationary phase, and 7:3 EA/*n*-Hx as mobile phase). The product was purified using silica-gel (60 mesh) column chromatography using the same mobile phase as in TLC. After solvent removal, the obtained oily light-yellow EgGMA monomer (~66% yield) was vacuum dried and stored in refrigerator until use.

### 2.3. Filler Modification

**Figure 1:**
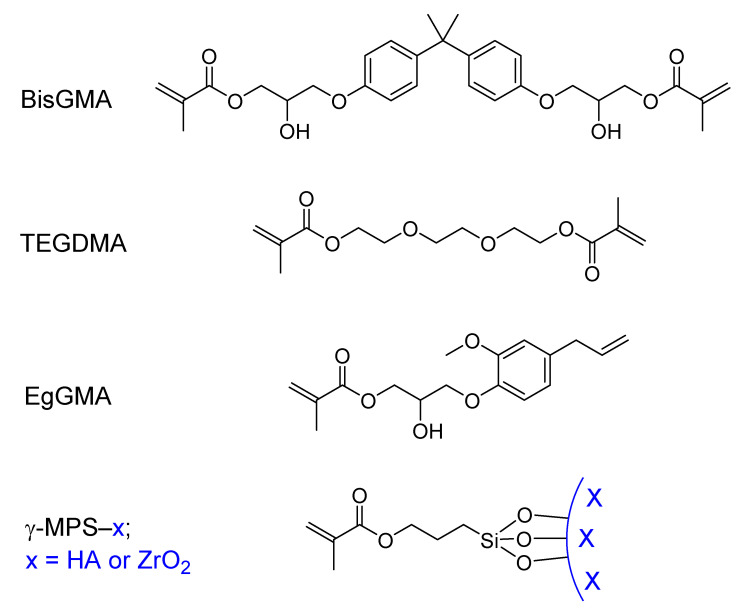
The chemical structure of BisGMA, TEGDMA, EgGMA and silanized particles.

Zirconia (ZrO_2_, <2 μm particle size) was silanized following a method described elsewhere [20] with slight modification. Thus, an excess amount of γ-MPS (0.6 g; 2 wt% with respect to the ZrO_2_) was hydrolyzed in 100 mL acetone with stirring for 2 h. Then, ZrO_2_ powder (30 g) was added slowly for 10 min and the suspension was left to stir magnetically overnight at room temperature. The suspension was filtered and washed using acetone and, to increase the interaction, the slurry was dried further in a laboratory oven at 100 °C for 3 h. The surface of hydroxyapatite (HA, powder, <5 μm particle size, surface area ≥ 100 m^2^/g) was modified with γ-MPS according to the procedure used by Lung et al. [21]. Briefly, a solution of 5 wt% of γ-MPS with respect to HA was prepared in 90 vol% ethanolic aqueous solution. The solution pH was adjusted to 4 by drops of acetic acid and hydrolyzed for 90 min with stirring at room temperature. To this solution, HA powder was added in batch, thoroughly dispersed, sonicated for 10 min, and left to stir overnight at room temperature. The silanized HA was filtered, washed with ethanol absolute to remove unreacted silanes and dried in an oven at 60 °C for 24 h. The chemical structures of the modified fillers are depicted in Figure 1, along with the base monomers (BisGMA, TEGDMA, and EgGMA).

### 2.4. Preparation of TBEg Composites

The monomer under investigation, EgGMA, was incorporated in resin-based dental composites at 0, 2.5, and 5 wt%, a protocol modified from Almaroof et al. [12]. Each composite (termed TBEg0, TBEg2.5, and TBEg5) consists of 35 wt% resin mixture (TBEg: TEGDMA, BisGMA, and EgGMA), 65 wt% filler (silanized HA and ZrO_2_), and 1.5 wt% (with respect to the total monomers) initiator system (CQ and DMAEMA), as given in Table 1. First, a mixture of equal masses of BisGMA and TEGDMA, representing the resin matrix, was prepared and, thereafter, partially replaced at 0.0, 2.5, and 5.0 wt% by EgGMA monomer to obtain the intended resin matrices. Photo-initiator was dissolved in the resin matrix and, subsequently, the fillers were added and manually mixed using stainless steel spatula under fairly dark light. The pastes were further homogenized using a dual asymmetric centrifugal mixing system (Speed Mixer TM DAC 150 FVZ, Hauschild and Co., Hamm, Germany) four times (for 1 min each, with 2 min rest in between) at 3000 rpm. The obtained pastes were molded and photo-polymerized using a LED curing light (Elipar S10, 3M ESPE, St. Paul, MN, USA) for 40 s (unless otherwise stated), as specified for various tests.

### 2.5. Degree of Vinyl Double-Bond Conversion

The degree of conversion (DC) of the polymerizable aliphatic bonds (C=C) into single bond (C–C) was calculated using Fourier transform infrared (FTIR) method, in reference to internal aromatic C=C bonds [22]. The specimens were compacted in a stainless-steel mold (5 mm diameter and 2 mm thickness, *n* = 5) and their FTIR spectra were recorded (termed uncured) using a Nicolet iS10 FTIR spectrometer (Thermo Scientific, Madison, WI, USA) equipped with an attenuated total reflection (ATR) accessory (diamond crystal) at 32 scans per spectrum and a resolution of 4 cm^−1^. Subsequently, the specimens were covered with plastic strips followed by glass slides to minimize the oxygen inhibition, light-cured for 40 s from both sides, and their ATR-FTIR spectra were recorded (cured). The calculation was carried out using Equation (1), taking into consideration the advantage of the change in the peak area (representing the mole fraction of the corresponding functional group) of the polymerizable C=C vinylic bond (A_1638_) with respect to that of the unaffected internal standard aromatic C=C bonds, denoted by A_1608_, before and after curing process.

(1) $$\mathrm{DC}\left(\%\right)=\left[1-\frac{\left(\frac{A_{1638}}{A_{1608}}\right)\mathrm{cured}}{\left(\frac{A_{1638}}{A_{1608}}\right)\mathrm{uncured}}\right]\times 100$$

### 2.6. Cytotoxicity Tests

#### 2.6.1. Cultured Fibroblasts

Gingival biopsies were harvested from patients who gave written informed consent according to the approval of the Institutional Review Board (IRB) for research on humans (Medical Faculty, King Saud University, Approval Number E-21-6412). Gingival fibroblasts (GFs) were cultured from explants of gingival tissue, and primary human gingival fibroblasts were cultured in DMEM medium (Dulbecco’s Modified Eagle Medium) (Sigma-Aldrich, St. Louis, MO, USA) supplemented with 10% fetal calf serum (FCS), 2 mM L-glutamine, antibiotics, and antimycotics at 37 °C in a humidified 5% CO_2_ incubator for further growth.

#### 2.6.2. Cell Viability Assay Using AlamarBlue^®^

Discs, 5 mm in diameter and 1 mm in thickness (*n* = 5), of TBEg composites were prepared in a stainless-steel mold and irradiated for 40 s using a dental LED-curing unit, as described above. The specimens were sterilized under an ultra-violet (UV)-light for 1 h on both sides before application.

Human gingival fibroblasts (HGFs) were cultured in 24-well plates, seeded at 5 × 10^4^ cells/well using HGF basic medium [23]. After 24 h of incubation, the specimen’s discs representing TBEg0, TBEg2.5 and TBEg5 were placed on the monolayer culture and incubated at 37 °C (5% CO_2_, 95% air) for 1, 4 and 7 days. The biocompatibility (cellular viability) of the HGFs was evaluated at the selected time points (1, 4, and 7 days) using alamarBlue^®^ (Biosource, Camarillo, CA, USA). Basically, alamarBlue is a water-soluble dye that used for quantifying in vitro viability of various cells. The active component of alamarBlue^®^ is resazurin, which is a stable redox indicator, nontoxic, blue in color, and non-fluorescent. Viable cells reduce resazurin to the weakly fluorescent resorufin, which can be detected by measuring fluorescence at 595 nm. Although a linear relationship between fluorescence and cell number is established, the level of fluorescence can be affected by both alteration in cell number (proliferation) and/or cell activity [24].

At the predefined time points (Day1, Day4 and Day7), the medium was refreshed. Thus, a fresh HGF basic medium containing 10% (by volume) alamarBlue^®^ was added to each well according to the manufacturer’s recommendations. After 1 h of incubation at 37 °C, triplicate, 200 μL samples from each well were taken into individual wells of a 96-well plate. Subsequently, the fluorescence intensity of each well-containing solution was measured with a Tecan fluorescent plate reader (Tecan, Männedorf, Switzerland) using an excitation wavelength of 540 nm and an emission wavelength of 570 nm. The florescence reading of negative controls based on cell-free samples were also determined, to assess any dye changes occurring in the absence of cells, which indicated no significant interaction between the dye and the composites under investigation (i.e., TBEg0, TBEg2.5 and TBEg5). Therefore, the fluorescence values were corrected using the value obtained for 10% alamarBlue^®^ solution in medium without cells seeded in the presence of TBEg’s discs as the negative control. The experiments were performed in triplicate and repeated twice.

### 2.7. Statistical Analysis

Data were analyzed using SPSS 21 (IBM Corp., Armonk, NY, USA). Variables were presented as mean ± standard deviation (SD) or standard error of mean (SeM). One-way analysis of variance (ANOVA) and Tukey post-hoc tests were used to analyze the significance of degree of conversion as well as the effect of materials and their period of interactions on cell viability. A *p*-value of <0.05 was considered significant.

## 3. Results and Discussion

### 3.1. Material Analysis

**Figure 2:**
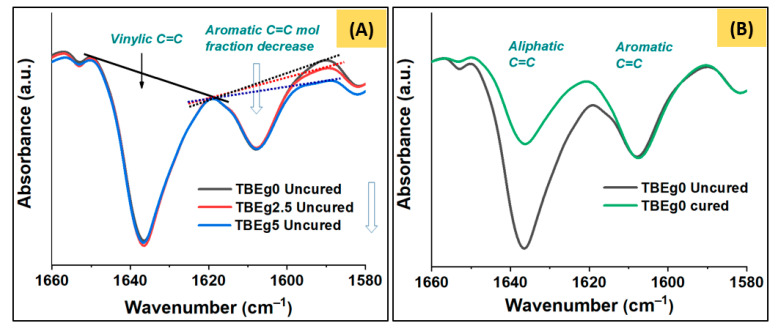
FTIR spectra on the range of C=C absorption (1660–1580 cm^−1^): (**A**) an illustration of uncured composites spectra showing the aromatic C=C mole fraction differences and (**B**) a representation of TBEg0 changes in aliphatic C=C due to photopolymerization process.

The target monomer, EgGMA, was synthesized by reacting eugenol with glycidyl methacrylate, using triphenylphosphine as a catalyst. Then, the product was column chromatography purified and characterized as reported previously [19]. Fillers, HA and ZrO_2_ and their combination ratio, were in accordance (with slight modification) with previous publication [12] in which eugenyl methacrylate was incorporated in experimental composites for core build–up restoration. After modification, the silanized-HA and -ZrO_2_ were used as-obtained, however, analysis indicated slight increased particle size. Experimental composites were basically designed to contain 35 wt% resin matrix and 65 wt% fillers and, to explore the effect of incorporation of EgGMA monomer, 0.0, 2.5 and 5.0% of the 35% matrix were replaced by EgGMA monomer. The composites thus termed TBEg0, TBEg2.5 and TBEg5 and the EgGMA percentages were equivalent to 0.0, 8.45 and 16.66 mol% with respect to a 100% resin mixture as shown in Table 1. The integrated area corresponding to the characteristic aromatic and aliphatic C=C bonds at 1608 and 1638 cm^−1^, respectively, of uncured composites TBEg0, TBEg2.5 and TBEg5 are shown in Figure 2. As the peak area is proportional to the mole fraction of the corresponding bond, an increase in the vinylic peak areas was observed due to the addition of EgGMA (Figure 2A). However, the ratio is more accurate for mole fractions comparison, and as many factors may affect the values of the integrated peak, comparison is only valid for relatively stable peaks with close absorptivity and must be from individual spectrum. Table 2 gives the calculated mole ratio, both theoretically and experimentally, from the resin initial composition and FTIR spectra, respectively. Such comparison supports the FTIR method for mole fraction calculation. The error% between theoretical and experimental calculation was found as less as 5%.

### 3.2. Degree of Conversion

The degree of double bond conversation (DC) of TBEg composites, measured directly after photo-curing step, are given in Table 3. The results indicate a decrease in DC for composites containing EgGMA (TBEg2.5 = 66.49% and TBEg5 = 58.03%) compared with that of the control (TBEg0 = 68.74%); however, no significant difference between TBEg0 (the control) and TBEg2.5 at *p* < 0.5. The reduction in the DC could be attributed to the reactivity difference between various vinylic moieties existing in the resin composites and some retained activity inhibitory effect of the Eg moiety. The delayed participation of allylic double bonds of the bi-functional nature (vinylic and allylic) EgGMA monomer, in which the allylic C=C is proven to be involved only in post-polymerization process, leading to DC development after 24 h [11]. Moreover, the post-polymerization is limited to the very first minutes after irradiation, as previously noted [25]. However, the obtained DC values were still above the minimum acceptable values for clinical use (>55%) [25,26]. Despite of viscosity enhancement, the DC was inversely affected, which is again mostly correlated with the EgGMA structural properties, which could retain some antioxidant properties on its aromatic and allylic functionalities [2,27].

### 3.3. Biocompatibility of TBEg Composites

**Figure 3:**
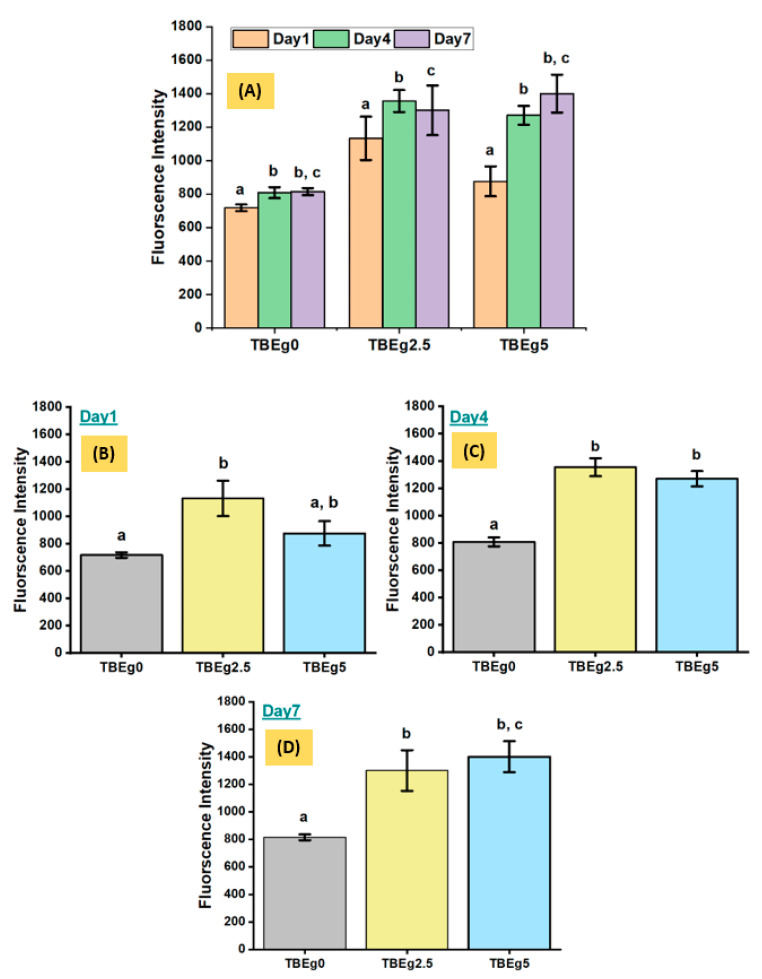
Cellular activity of HGFs cultured with different composite discs (TBEg0, TBEg2.5 and TBEg5) for 1, 4 and 7 days, after 1 h incubation in alamarBlue^®^. (**A**) Viable cells count for different composites; different lowercase letters indicate statistically significant differences within each group at *p*–value of 0.05. (**B**–**D**) Effect of TBEg type on the activity of HGFs at the time point of 1, 4 and 7 days, respectively; different lowercase letters indicate statistically significant differences between composite (TBEg0, TBEg2.5 and TBEg5) (*p* < 0.05).

The cell viability test of the HGFs using alamarBlue^®^ assay indicates that the HGFs remained viable throughout the culture time (7 days), with increasing cellular viability from Day1 to Day7 in groups TBEg0 and group TBEg5, and this difference is statistically significant (Figure 3). HGF culture with the TBEg’s discs for 24 h (Day1) were viable; however, the viability in group TBEg2.5 was significantly higher than that in the control group (TBEg0) (*p* < 0.01). HGFs cultured with group TBEg5 discs exhibited cell viability comparable to that with the control group. At Day4, HGFs cultured with TBEg discs in group TBEg0 and group TBEg5 showed increased cellular viability compared with that in Day1, and this increase is statistically significant (*p* < 0.000). Despite the increase in the cellular activity compared with that in Day1, HGFs cultured with eugenol discs in group TBEg2.5 and group TBEg5 showed significantly higher cellular viability when compared with that of the control group (*p* < 0.000). The same pattern was observed in Day7, wherein all the tested groups exhibited significantly higher cellular viabilities compared with that of the control group at Day7 (*p* < 0.002) (Figure 3).

Overall, in the biocompatibility test presented in this study, the cellular viability was high in all tested groups, with observed increase in culture with respect to time (Figure 3). This result indicates that all materials used were fairly biocompatible with no drastic cytotoxicity effect observed. Compared with the control group (TBEg0), both TBEg2.5 and TBEg5 groups showed better cellular viability at Day4 and Day7. This could be related to the compositions of the tested specimens, in which the traditional base resin (BisGMA/TEGDMA) was partially replaced at 2.5 (TBEg2.5) and 5 wt% (TBEg5) by EgGMA monomer (TBEg0, the control) in the composites, which contained primarily 65 wt% filler (Table 1). This behavior, and thus the toxicity of the resin components in each composite, could be explained based on the chemical structure; however, the toxicities of the conventional monomers (BisGMA and TEGDMA) have already been reported [28]. Moreover, the biocompatibilities and toxicities of eugenol and its numerous derivatives have been investigated [7,9].

The leached-out unreacted monomers are the main cause of cell cytotoxicity due to the oxidative stress to the cell (oxidative stress is a case of imbalance between free radical production and their degradation driven by antioxidant systems) [29]. Assuming that the amounts of the leachable materials are proportional to the component ratio in the composite, leachable amounts for BisGMA and TEGDMA will decrease, whereas that for EgGMA will increase (EgGMA constitutes about 8 and 17 mol% of the total resin mixtures in the experimental composites, TBEg2.5 and TBEg5, respectively). Although the antioxidant activity of Eg derivatives is less than that of free eugenol molecules, its effectiveness, to some extent, has been reported to be retained, functioning through the allylic group [30]. Therefore, the increased viability with respect to the amount of EgGMA may be attributed to this phenomenon. The significantly high cell viability at Day1 may be due to the higher amounts of antioxidants released from TBEg2.5 and TBEg5 in the first day compared to the control, whereas at Day4 and Day7, the functionality of the immobilized EgGMA is mostly the responsible factor for viability, which could be proven by its higher value for TBEg5 compared to that for TBEg2.5

## 4. Conclusions

The incorporation of EgGMA monomer as a new immobilizable derivative of eugenol within the matrix components of resin-based dental composites is a promising approach to enhance restorative martials properties including biocompatibility. The experimental findings indicate insignificant difference in degree of conversion of TBEg2.5, in which 8.45 mol% of the matrix is EgGMA, compared to the conventional composite (TBEg0 (control), EgGMA-free). More interestingly, composites overall biocompatibility was remarkably improved by addition of EgGMA. Thus, it could be concluded that, although EgGMA slightly suppresses the polymerization noticeably at high concentration, composites containing EgGMA are still safer than the control. Additionally, EgGMA incorporation in low quantities may retain eugenol desirable biological features. However, to deliver such material into future applications, more investigations are essential, that is in term of physicochemical, mechanical and antimicrobial properties of EgGMA–incorporating composites.

## Figures and Tables

**Table 1:** Compositions of the experimental resin matrices.

Composite	Monomers (%)					
	TEGDMA		BisGMA		EgGMA	
	wt%	mol%	wt%	mol%	wt%	mol%
TBEg0	50.00	35.84	50.00	64.16	0.00	0.00
TBEg2.5	46.43	32.81	46.43	58.74	7.14	8.45
TBEg5	42.86	29.87	42.86	53.47	14.29	16.66

Note: each composite contains (by wt%) 35% resin mixture, 65% filler mixture (HA/ZrO_2_, 4:3 wt/wt), and 0.5 CQ and 1.0 DMAEMA initiation system with respect to the total monomer. Abbreviations: BisGMA, bisphenol A-glycidyl methacrylate; CQ, camphorquinone; DMAEMA, *2*-(*N*,*N*-dimethyl amino) ethyl methacrylate; EgGMA, eugenyl–glycidyl methacrylate; HA, hydroxyapatite; TEGDMA, triethylene glycol dimethacrylate; ZrO_2_, zirconium dioxide.

**Table 2:** Mole ratio between the aliphatic (vinylic) and aromatic C=C bonds calculated theoretically from the composition and experimentally from FTIR spectra. For easy comparison, values were normalized to the control (1.00) in both cases.

Composite	Theoretical (Composition)		Experimental (Observed, FTIR)		Error%
	Value	Normalized to TBEg0	Value	Normalized to TBEg0	
TBEg0	1.790	1.000	2.950	1.000	0.0
TBEg2.5	2.048	1.144	3.406	1.155	1.0
TBEg5	2.348	1.312	3.649	1.237	−5.7

**Table 3:** The mean and standard deviation (SD) of TBEg’s composites degree of conversion (DC), (*n* = 5).

Formulation	TBEg0		TBEg2.5		TBEg5	
DC %	Average	SD	Average	SD	Average	SD
	68.74	0.77 ^a^	66.49	1.851 ^a^	58.03	1.238 ^b^

Note: within the raw, different lowercase letters (a, b) indicate a statistically significant difference at *p*-value < 0.05.

## Data Availability

Data that support the findings of this study are included in the article.
